# Muscle Oxygen Saturation Breakpoints Reflect Ventilatory Thresholds in Both Cycling and Running

**DOI:** 10.2478/hukin-2022-0054

**Published:** 2022-09-08

**Authors:** Andri Feldmann, Linda Ammann, Flurin Gächter, Marc Zibung, Daniel Erlacher

**Affiliations:** 1 Institute of Sport Science, University of Bern, Bern, Switzerland

**Keywords:** NIRS, anaerobic threshold, tissue oxygen saturation, lactate threshold

## Abstract

Pulmonary gas exchange analysis was compared to changes in muscle oxygen saturation as measured by near-infrared spectroscopy. First, ventilatory thresholds determined by common gas exchange analysis and breakpoints in muscle oxygen saturation were assessed for agreement during exercise with increasing intensity. Secondly, the relationship between muscle oxygen saturation as a surrogate for local oxygen extraction and peak oxygen uptake was assessed. In order to lend robustness to future NIRS testing on a broader scale, considering its potential for simple and cost-effective application, the question of a running versus a cycling modality was integrated into the design. Ten participants, of whom five were recreationally trained cyclists and five recreationally trained runners, were tested; each during a cycling test and a running test with increasing intensity to voluntary exhaustion. Muscle oxygen saturation and pulmonary gas exchange measurements were conducted. Bland-Altman analysis showed a moderate degree of agreement between both muscle oxygen saturation breakpoint 1 and muscle oxygen saturation breakpoint 2 and corresponding ventilatory threshold 1 and ventilatory threshold 2, for both cycling and running disciplines; generally speaking, muscle oxygen saturation breakpoints underestimated ventilatory thresholds. Additionally, a strong relationship could be seen between peak oxygen uptake and the minimally attained muscle oxygen saturation during cycling exercise. Muscle oxygen saturation measured using NIRS was determined to be a suitable method to assess ventilatory thresholds by finding breakpoints in muscle oxygen saturation, and muscle oxygen saturation minimum was linked to peak oxygen uptake.

## Introduction

Exercise threshold testing is a cornerstone of applied exercise physiology. While the gold standard for this type of testing remains pulmonary gas exchange analysis, alternative methods are available. One such method involves near-infrared spectroscopy (NIRS) to measure muscle oxygenation in the working muscle. While this approach is not common practice among performance diagnosticians, it has been well documented in recent years and has proven to yield reliable results that reflect the pulmonary gas exchange analysis (Bhambhani et al., 1997; Fontana et al., 2015; Grassi et al., 2003; Spencer et al., 2012). Mechanistically this makes sense, as NIRS measurements have been shown to accurately reflect venous oxygen saturation (Mancini et al., 1994).

Muscle oxygenation as measured by NIRS, and thereby the relationships mentioned above, involve a series of terminological and methodological differences. Firstly, the most cost-effective and commonly applied NIRS technique, i.e., continuous-wave (Perrey and Ferrari, 2018), provides arbitrary values for concentrations of oxyhemoglobin and oxymyoglobin, and deoxyhemoglobin and deoxymyoglobin, both of which are often identified with differing acronyms. This discrepancy is outlined in a recent review by Barstow (2019). For this reason, we will apply the nomenclature recommended, oxy[heme] and deoxy[heme] to identify oxy- and deoxyhemoglobin and myoglobin, respectively. This also identifies methodological differences, if assessing muscle oxygenation, is oxy[heme] or deoxy[heme] dynamics relevant? This is inconsistent in the literature and shows differing results. Furthermore, which of the two variables is more relevant physiologically, or is there a third or better option? Combining the two variables, oxy[heme] and deoxy[heme], a third option is available which is muscle oxygen saturation (SmO_2_). While in his review Barstow (2019) does identify this nomenclature as well and proposes to match both tissue oxygen saturation and muscle oxygen saturation under the common term StO_2_, we disagree and propose to leave the term as SmO_2_. The sole reason being, t would imply homogenous tissue of multiple layers, whereas m implies specifically an attempted isolation of the muscle tissue (Feldmann et al., 2019b). The two concepts are therefore not the same. SmO_2_ offers a third NIRS derived variable to assess pulmonary gas exchange and one that is novel to this investigation. What is the relationship between SmO_2_ and pulmonary gas exchange?

Muscle oxygenation dynamics during incremental exercise have been documented in four phases (Bhambhani, 2004). First, there is an initial phase where an increase and or a plateau in muscle oxygenation can be seen. This is followed by phases two and three which create a segmented-linear function to a minimum muscle oxygenation plateau value with exhaustion (Bhambhani, 2004; Spencer et al., 2012). Phase four is the hyperemia phase with an overshoot of muscle oxygenation when compared to baseline. This predictive pattern has generated comparisons between muscle oxygenation dynamics and ventilatory thresholds (VT). Fontana et al. (2015) conducted a large study with 118 males who all completed incremental cycling tests and found a good agreement between NIRS derived breakpoints using deoxy[heme] and the respiratory compensation point. Van der Zwaard et al. (2016) found a similar agreement, however, in this case finding agreement between the difference of oxy[heme] and deoxy[heme] and VT1 rather than deoxy[heme] and the respiratory compensation point. Racinais et al. (2014) using both oxy[heme] and deoxy[heme] signals found a moderate relationship between NIRS dynamics and VT. As can be seen, there is some discrepancy as to what physiological thresholds are exactly represented by which characteristics of the NIRS signal.

When considering which variable or combination of variables to use to determine NIRS derived breakpoints, a discussion of physiological legitimacy should be held. How reliable are specific NIRS variables to determine the relevant physiology? This has been discussed, highlighting, for example, the fact that oxy[heme] is highly sensitive to blood volume changes in superficial tissue and therefore offers a poorer indicator of the relevant physiology (Quaresima and Ferrari, 2009). For this reason, numerous authors, mentioned earlier, have applied deoxy[heme] to their evaluations. However, deoxy[heme] is also subject to changes in total[heme] at the local level, and deoxy[heme] only represents oxygenation changes accurately if total[heme] remains stable (Quaresima and Ferrari, 2009). This is highly suspect during whole-body exercise. For this reason, it is recommended to use a combined value including both oxy[heme] and deoxy[heme] to better represent the influence of blood volume changes (Quaresima and Ferrari, 2009). SmO_2_ as presented in this study accomplishes this task, as SmO_2_ is subject to both oxy[heme] and deoxy[heme] changes. Our first goal for this study was, therefore, to examine the relationships between VT1 and VT2 and NIRS, specifically SmO_2_ breakpoints (BP), including the two most popular endurance testing modalities, i.e., running and cycling.

Secondly, change in the NIRS derived muscle oxygenation signal has been directly tied to changes in local muscular oxygen extraction and utilization (Grassi and Quaresima, 2016) and therefore tightly linked to arterio-venous difference at rest and during exercise (Ferreira et al., 2005). For this reason, numerous studies have demonstrated a correlation between VO_2_ and changes in muscle oxygenation (Hiroyuki et al., 2002; Okushima et al., 2016; Wang et al., 2012). Considering the two corresponding Fick principles (Wagner, 2011), the Fick principle of convection and Fick’s law of diffusion, this relationship is appropriate. Investigating maximal oxygen extraction in terms of minimally attainable SmO_2_ (SmO_2_min) in different exercise modalities and how it reflects VO_2_peak is an interesting course of action keeping fitness assessments and predictions in mind. Our second goal was, therefore, to examine the relationship between SmO_2_min and VO_2_peak.

## Methods

### Participants

Fourteen participants took part in the study. Of the fourteen, four participants were removed from the analysis because of adipose tissue thickness (ATT); of the remaining ten participants six were males (29.3 ± 6.6 years; body mass: 74.8 ± 9.5 kg; body height: 1.79 ± 0.04 m) and four were females (age: 26.5 ± 4.2 years; body mass: 55.5 ± 3.9 kg; body height: 1.65 ± 0.05 m). ATT limits were set to half of the emitter-detector distance, approximately 15 mm (Feldmann et al., 2019a). Five participants were recreationally active cyclists (4 males, 1 female) with a minimum of three years of cycling-specific training experience and the other five were recreationally active runners (2 males, 3 females) with a minimum of three years of running-specific training experience. All participants were in good health, non-smokers, and unmedicated. Participants were informed of the study design and the physical tasks ahead of time and written informed consent was obtained in advance. The study was carried out in accordance with the 1964 Declaration of Helsinki. The protocol was approved by the ethics committee of the local Faculty of Human Sciences.

### Measures

Gas exchange was measured continuously (breath-by-breath) using the Oxycon Jaeger (Vyaire Medical GmbH, DE). SmO_2_ was measured using NIRS sensors placed on the vastus lateralis muscle of the right and the left leg two-thirds between the anterior superior iliac spine and the lateral side of the patella. The sensors were fixed in place using medical adhesive tape (Hypafix; BSN Medical, DE) and were then covered with a compatible commercially available light shield to eliminate possible ambient light intrusion. Certain exercise modalities or physiological conditions can result in differences in right and left limb muscle oxygenation dynamics (Hesford et al., 2012; van Hooff et al., 2018). To ensure that the leg choice did not affect the evaluation of thresholds both legs were measured and compared. A commercially available continuous-wave NIRS device was used (Moxy Monitor; Fortiori Designs LLC, US) to measure SmO_2_. The device uses four wavelengths (680,720,760 and 800 nm) to assess absorbency via modified Beer-Lambert resulting in a relative concentration of SmO_2_ as percent in the following equation: oxy[heme] / (oxy[heme] + deoxy[heme]) = SmO_2_. The device detectors are spaced at 12.5 and 25 mm from the emitter. The sampling rate was set at a default mode which samples the four wavelengths over 80 cycles for averaged output every two seconds (0.5 Hz) and gathered using the SWINCO NIRS software (Swinco AG, CH). The Moxy Monitor has been assessed for reliability and validity in a series of publications (Crum et al., 2017; Feldmann et al., 2019b).

### Design and Procedures

All participants took part in two maximum effort incremental step tests with one-week between the evaluations at the same time of day. One test was completed on a cycling ergometer (Ergoline 800s) and the second on a treadmill (Woodway PPS 55sport). Participants were randomly selected to either start with the run or the cycle test. Pulmonary gas exchange and NIRS measurements were taken during exercise.

The run test started with a standard 5-min warm-up at 3.0 km/h for females and 3.5 km/h for males both with a 7% incline. The test protocol used 30 s increments with speed increases of 0.5 km/h at a steady incline of 7% starting at 6.0 km/h for females and 7.0 km/h for males. The test was considered maximum and participants were motivated to maximize exertion to voluntary exhaustion. The cycle test started with a standard 5-min warm-up at 50 watts for females and 100 watts for males. The test protocol used 60 s increments with power increases of 25 watts with starting watts of 50 for females and 100 for males. The allowable cadence range was set between 80100 rpms. Again, the test was considered maximum, and participants were motivated to maximize exertion to voluntary exhaustion. Guidelines for the test protocol were taken from the Swiss Olympic Performance Diagnostics manual (Maier et al., 2016) for VO_2peak_ testing.

### Statistical Analysis

VT1 and VT2 were determined by an expert using the standard 9-panel plot for cardiopulmonary exercise testing (CPET) (Dumitrescu and Rosenkranz, 2017). VT1 was identified as a non-linear increase in VCO_2_ to VO_2_ above a ratio of 1:1. In addition, VT1 should be matched with an increase in the ratio of VE/VO_2_ without an increase in VE/VCO_2_ and an increase in PETO_2_ without a decrease in PETCO_2_. VT_2_ was identified as a non-linear increase in VE to VCO_2_. Additionally, an increase in the ratio of VE/VCO_2_ and a decrease in PETCO_2_ should be seen. In order to determine BP1 and BP2, a completely automated segmented regression analysis to minimize the sum of square residuals was used for the SmO_2_ data; analogous to the method applied by Spencer et al. (2012). The segmented regression analysis applied four-knot points to identify three of the four phases of SmO_2_ described in the introduction and therefore two SmO_2_ BP. The first and last knots identify the start and the end of the test, and the two knots between identify BP1 and BP2, respectively. The first 60 s of every test were ignored in the segmented regression, as the rather abrupt starts (specifically in the running protocol) result in abrupt changes in the NIRS signal. To assess agreement between VT1 and BP1, and VT2 and BP2 Bland Altman plots were established comparing heart rates at the given thresholds. The heart rate was chosen as the common practice variable for training zoning. Upper and lower limits of agreement were set at 1.96 SD with the recommended CI (Bland et al., 1986). The same was done to assess agreement between the left and the right leg for BP1 and BP2. VO_2peak_ and SmO_2min_ were evaluated using Pearson’s product-moment correlation to determine their relationship. Additionally, the means and standard deviation of the different participant groups (cyclist and runner) and tests (cycling and running) were calculated to determine effect sizes and plotted. Statistical computations were performed using Microsoft Excel for Windows (Version 16.0.4738.1000) and MathWorks Matlab for Windows (Version 9.3.0.713570 R2017b).

## Results

**Figure 1:**
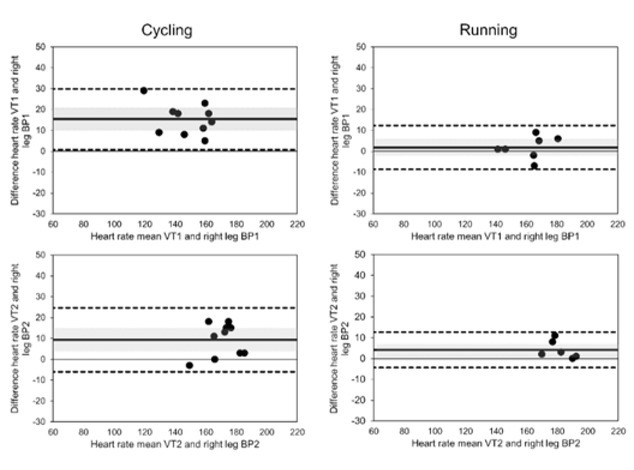
Two different time courses of SmO2 (o) with increasing performance to voluntary exhaustion. Both charts presented are cycling test results. The dashed vertical lines represent left leg BP1 and BP2, respectively. The red lines represent the linear regression lines that dictate BP1 and BP2. The gray area represents the power time course and the solid black line the heart rate time course.

The time course of SmO_2_ reflects expected changes as a result of increasing performance (Figure 1) with three phases as described by Bhambhani (2004). The first phase at low-intensity SmO_2_ shows slightly positive or plateauing trends from baseline. In some cases, the start of exercise was met with a strong decrease in SmO_2_ followed by a plateau. This phenomenon was seen to a greater degree in running with six of ten participants showing this phenomenon to a severe degree, while none of the cycling tests showed it to a severe degree. For this reason, the first 60 s of every test were not considered for the BP analysis. The greater relative change in performance demand from the warm-up to test exercise is considered to be the major contributor. The second phase is identified by a continuous drop in SmO_2_ with increasing performance. The third phase is identified by a leveling off or a positive inflection in SmO_2_ until test cessation. A fourth phase described by Bhambhani (2004) is the expected hyperemia response, and it was also seen, but not analyzed.

**Figure 2:**
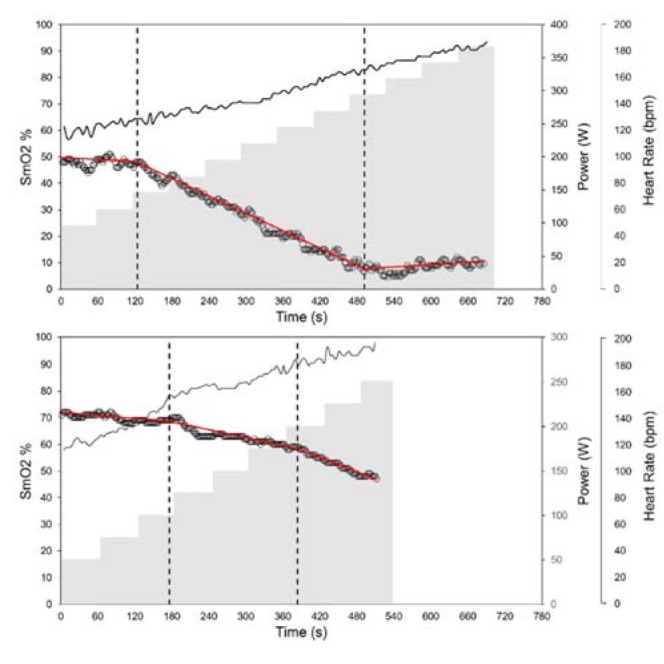
Figure 2 Bland-Altman plots of SmO2 BP and VT for both cycling and running tests. The solid line identifies the mean bias (MB) and dashed lines the upper and lower limits of agreement at ±1.96 SD. The gray shaded area represents the 95% CI for MB. For a few of the tests a clear VT2 could not be identified, as seen in the VT2 Bland-Altman plots; see text for details.

The deflection points between phase 1 and phase 2 and between phase 2 and phase 3 were identified as BP1 and BP2, respectively. While numerous tests showed the expected pattern described, a large number failed to show the positive deflection or attenuation in phase 3. Instead of a leveling-off, a second negative deflection point can be observed that results were in a further decrease in SmO_2_ as seen in Figure 1. This pattern is a closer representation of O_2_Hb findings by Racinas et al. (2014). Nonetheless, this deflection point was identified as BP2. In total, of the 10 cycling tests, BP2 showed a positive inflection in seven tests and negative inflections in three tests. For running, BP2 showed a positive inflection in two tests and a negative one in five tests. For running, three tests identified only one clear BP. Using BP to identify VT appears to show a certain degree of consistency, with a moderate degree of agreement (Figure 2). BP1 for cycling appears to underestimate VT1 (Mean Bias (MB): 15.4 ± 7.4 bpm); this is not the case for running which shows a good agreement (MB: 1.8 ± 5.6 bpm). BP2 and VT show a moderate degree of agreement for both cycling and running; in both cases, BP2 underestimates VT2 (cycle MB: 9.3 bpm ± 7.8, run MB: 4.2 bpm ± 7.2). Time points of thresholds showed similar agreement to the heart rate; VT1 (cycle MB: 70.6 ± 64.0 s, run MB: 10.4 ± 49.1 s), VT2 (cycle MB: 5.8 ± 59.2 s, run MB: 5.0 ± 28.3 s). In terms of power or speed, this represents in both cases the range of approximately one performance step.

**Figure 3:**
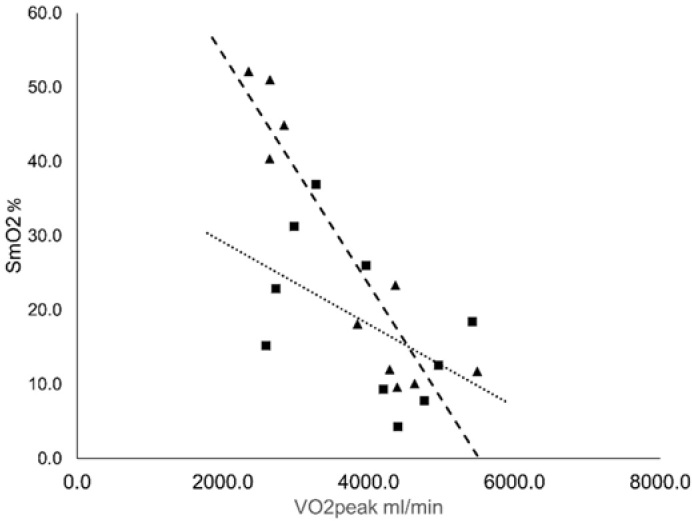
Relationship between minimally attained SmO_2_ value and VO2_peak_ at voluntary exhaustion. The dotted line illustrates the line of the best fit for running (■); R2 = 0.266. The dashed line illustrates the line of the best fit for cycling (▲); R2 = 0.8512.

VO_2peak_ for each test was correlated to SmO_2min_ value at the cessation of the test (Figure 3). A strong correlation existed between SmO_2min_ and VO_2peak_ for cycling (R^2^ = 0.8512). This was not the case for running, which showed a substantially weaker relationship (R^2^ = 0.266).

**Figure 4:**
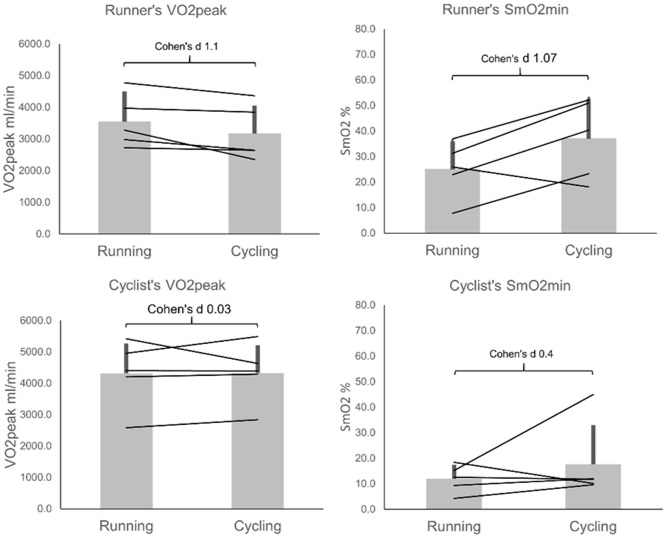
Mean and standard deviation of cycling and running test VO_2peak_ and SmO_2min_ for both the cyclists specialised group and the running specialised group..

Examining VO_2peak_ and SmO_2min_ between cyclists and runners, a clear tendency can be seen. Runners showed higher VO_2peak_ in their trained discipline (Figure 4) as expected. Cyclists showed slightly higher VO_2peak_ in cycling, but the difference was negligible. The cycling group overall presenteded itself as fitter with higher VO_2peak_ in both disciplines. In accordance with the correlation between VO_2peak_ and SmO_2_, runners showed lower SmO_2min_ during running than cycling, reflecting their difference in VO_2peak_. The smaller difference in VO_2peak_ seen in cyclists was also reflected by a smaller difference between cycling and running SmO_2min_; as can be seen, a single athlete presented themselves as an outlier in showing a large difference in SmO_2min_ for cycling and running. This should be considered when assessing the data.

## Discussion

As shown in the results and mentioned in the introduction, there is a moderate agreement between VT and BP. Generally speaking, for both VT1 and VT2, BP appears to underestimate the threshold, implying the break comes earlier than VT would anticipate. Considering the NIRS measurement tool, at the source of O_2_ exchange in the muscle this is a reasonable observation. Nonetheless, practical estimations of both VT1 and VT2 can be made using BP. However, when assessing likeness between NIRS derived thresholds and VT in published research stark contrasts can be noted between SmO_2_ and other NIRS variables. Numerous papers have identified BP to correspond with VT1 and or with lactate thresholds (Bhambhani et al., 1997; Grassi et al., 1999; Raleigh et al., 2018; van der Zwaard et al., 2016), whereas others indicated greater similarities between BP and VT2 (Fontana et al., 2015; Inglis et al., 2017).

None of the mentioned studies have identified two BP to correspond with VT1 and VT2. The study by Racinais et al. (2014) attempted to identify both VT1 and VT2 using O_2_Hb and HHb, but with limited success. A reason for these discrepancies might be in the approach taken to determine VT (Shimizu et al., 1991). However, more likely is the discrepancy to determine BP, as this process lacks standardization. The lack of standardization is partly due to the statistical and expert analysis to assess BP, but also the way in which the raw NIRS signal is interpreted. BPs are identified in most cases either as deflections in the oxy[heme] and deoxy[heme] difference or in deoxy[heme] alone. Only Raleigh et al. (2018) and Spencer et al. (2012) used a saturation, similar to SmO_2_ outlined in this study to determine BP. These variations are likely responsible for a considerable amount of the discrepancy. The strength of SmO_2_ may be identified herein in accuracy to identify VT and in identifying both VT1 and VT2. Two factors could be responsible for this outcome. First is that SmO_2_ accounts for both changes in deoxy[heme] and oxy[heme]. Second, the reason may be because of using SmO_2_ rather than a more general StO_2_, in this way applying a NIRS method that isolates the relevant muscle tissue layer, rather than a more homogenous tissue layer as reported in the studies by Spencer et al. (2012) and Raleigh et al. (2018). Assuming that muscle physiology is relevant for BP, changes in superficial tissue should be subtracted from an analysis and the focus set on the muscle layer.

While in this study right and left vastus lateralis BP agreed, the muscle probe site is highly relevant when discussing the assessment of BP during incremental exercise. Wang et al. (2012) showed differences in BP for the vastus lateralis and gastrocnemius during incremental cycling tests. Both muscles were considered to be useful to identify aerobic capacity, and both underestimated VT similar to this data collection. The determined BP was considered to be significantly different from one another. The same is true for NIRS patterns of the vastus lateralis and gastrocnemius during incremental running exercise (Hiroyuki et al., 2002). Further data in this area need to be collected to assess overlap between the muscle site probe and BP to determine effectiveness for standard exercise testing. However, variation in muscle activation and biomechanics can help understand differences in SmO_2_ dynamics. Changing muscle activity as a result of changing gait patterns with increasing speed alone (Kyrolainen et al., 1999) could account for running differences; the same is true for the cycling position (Li and Caldwell, 1998).

A point of interest when reviewing the time course of SmO_2_ during incremental exercise, even though there is an expected three-phase pattern, is that this pattern is by no means consistent. Rather there are two more likely expected patterns, both with a negative deflection point which can be correlated to VT1. However, the second defection point can be either positive or negative. Understanding the mechanism of the differentiating deflection points may be of diagnostic interest. One explanation is obvious as SmO_2_min values approaching zero can only have a positive deflection point response, and therefore perhaps the differences in a positive or negative deflection have solely to do with an approach towards 0% SmO_2_. For the majority of cases in this study, this does seem to be the case with SmO_2min_ values approaching 0% showing this positive deflection, although it is not the case for all participants. Nonetheless, achieved SmO_2min_ and perhaps in combination with a positive or negative deflection point, could infer cardiorespiratory fitness as pointed out in the data that SmO_2min_ and VO_2peak_ have a strong correlation (Hiroyuki et al., 2002; Wang et al., 2012). SmO_2_ may present itself as an effective way to assess components of cardiorespiratory fitness, with the focal point being on the arterio-venous difference of specific muscles. The fact that SmO_2min_ and VO_2peak_ have a stronger correlation for cycling than for running further underscores the insight into arterio-venous difference that NIRS offers, as the vastus lateralis is a major contributor to systemic VO_2_ for cycling and lesser so for running (Millet et al., 2009).

Performance diagnostics is a standard procedure in high-level athletics. Limited largely by technological and equipment-related factors to gather relevant physiological information, performance diagnostics has been, in large part, reserved for laboratory data collection. However, with rapid development in wearable and mobile technologies, it is almost a certainty that aspects of performance diagnostics will be exported to in-situ assessments. This allows coaches and trainers to assess athletes repeatedly and in-training or in-event situations. This is especially true for NIRS and SmO_2_ measures as a non-invasive measurement analogous to a heart rate monitor in terms of data collection. While this paper, supported by the numerous citations which come to a similar conclusion, suggests that NIRS is a suitable alternative to gold standard threshold testing, we believe that any test form is highly subject to protocols chosen to elicit results. For this reason, when automating results or assessing data it is important to engage with the specific protocol used and the sports discipline assessed. For example, when comparing running and cycling one needs to be cautious in the assessment of general fitness using SmO_2_ as an absolute. The muscle site and a sports discipline chosen to be assessed will affect these measurements. Additionally, ATT is a source of concern that should be carefully measured to ensure accuracy (Craig et al., 2017). The ATT question also gives rise to the question of sex differences. As shown by McManus and colleagues (McManus et al., 2018), sex differences can be seen when using NIRS. However, it must also be noted that in this study the ATT differences between the female and male groups were large, and this alone could be the defining difference. While muscle mass and quality may also be factors which would separate males and females when using NIRS, this would most likely be related to training status and age more so than biological sex. When assessing the two distinct patterns mentioned earlier for the SmO_2_% time course for biological sex and ATT, no clear conclusion can be reached. Nonetheless, there appears to be a slight skew towards higher ATT yielding the two negative deflection points. Biological sex, however, did not appear to have an influence on this when taking ATT into consideration.

## Conclusion

The results of this study indicate that NIRS derived_2_ BP are suitable to determine both VT1 and VT2. Additionally, SmO_2min_ as a variable may present itself as a useful indicator of cardiorespiratory fitness as it correlates with VO_2peak_ depending on the exercise modality. With the NIRS device placed on the vastus lateralis, regardless of the right or the left leg, a cycling or a running exercise modality, BP and VT remained consistent. This increases certainty when applying only a single device to perform diagnostics. The findings of this study further emphasize the usefulness of NIRS based SmO_2_ measures to assess physical fitness and established training zones, as a valid alternative to pulmonary gas exchange methods.
